# Preferential digestion of PCNA-ubiquitin and p53-ubiquitin linkages by USP7 to remove polyubiquitin chains from substrates

**DOI:** 10.1074/jbc.RA118.005167

**Published:** 2019-01-15

**Authors:** Yuji Masuda, Rie Kanao, Hidehiko Kawai, Iwao Kukimoto, Chikahide Masutani

**Affiliations:** From the ‡ Department of Genome Dynamics, Research Institute of Environmental Medicine, Nagoya University, Furo-cho, Chikusa-ku, Nagoya 464-8601, Japan,; § Nagoya University Graduate School of Medicine, 65 Tsurumai-cho, Showa-ku, Nagoya 466-8550, Japan,; ¶ Graduate School of Biomedical and Health Sciences, Hiroshima University, 1-2-3 Kasumi, Minami-ku, Hiroshima 734-8553, Japan, and; ‖ Pathogen Genomics Center, National Institute of Infectious Diseases, 4-7-1 Gakuen, Musashi-murayama, Tokyo 208-0011, Japan

**Keywords:** deubiquitylation (deubiquitination), DNA damage response, enzyme kinetics, p53, proliferating cell nuclear antigen (PCNA)

## Abstract

Ubiquitin-specific protease 7 (USP7) regulates various cellular pathways through its deubiquitination activity. Despite the identification of a growing number of substrates of USP7, the molecular mechanism by which USP7 removes ubiquitin chains from polyubiquitinated substrates remains unexplored. The present study investigated the mechanism underlying the deubiquitination of Lys^63^-linked polyubiquitinated proliferating cell nuclear antigen (PCNA). Biochemical analyses demonstrated that USP7 efficiently removes polyubiquitin chains from polyubiquitinated PCNA by preferential cleavage of the PCNA-ubiquitin linkage. This property was largely attributed to the poor activity toward Lys^63^-linked ubiquitin chains. The preferential cleavage of substrate-ubiquitin linkages was also observed for Lys^48^-linked polyubiquitinated p53 because of the inefficient cleavage of the Lys^48^-linked ubiquitin chains. The present findings suggest a mechanism underlying the removal of polyubiquitin signals by USP7.

## Introduction

Ubiquitination regulates a wide variety of cellular processes such as proteasome-dependent proteolysis, autophagy, immune responses, DNA damage responses, and gene expression. Modification of target proteins by ubiquitination is catalyzed by target-specific ubiquitin ligase(s) (E3), which act in combination with ubiquitin-conjugating enzyme(s) (E2) specific for individual E3s. Each E3 specifically modifies target protein(s) by catalyzing their monoubiquitination or polyubiquitination with differently linked ubiquitin chains, which determine the fate of the modified proteins (1–3).

Deubiquitinating enzymes remove ubiquitin moieties from ubiquitinated proteins (4). The ubiquitin-specific protease 7 (USP7) belongs to a subgroup of deubiquitinating enzymes and regulates various cellular pathways by catalyzing the removal of ubiquitin chains (5). USP7 contains an N-terminal tumor necrosis factor receptor-associated factor-like (TRAF)^2^ domain, a central catalytic USP domain, and five consecutive ubiquitin-like (Ubl) domains (Ubl1–Ubl5) at its C terminus (6). The TRAF domain recognizes a P/A/E*XX*S motif in target proteins, which include p53 and MDM2 (5, 7–9). The Ubl1 and Ubl2 domains are stably connected and required for interaction with the other set of target proteins containing K/R*X*K*XXX*K motifs (5, 10–13). The remaining stably connected Ubl4 and Ubl5 regions and the C-terminal tail are required for the active conformation of USP7 (5, 10, 13, 14).

A growing number of substrates of USP7 have been reported, and deubiquitination reactions such as the deubiquitination of p53 have been examined *in vitro* (5, 10, 15–17). However, the results of biochemical analyses of USP7 to determine its substrate specificity are controversial. One report demonstrated that USP7 can remove a single ubiquitin moiety from a model substrate, monoubiquitinated CycB-Lys^60^, but cannot remove or digest Lys^48^-linked ubiquitin chains from polyubiquitinated CycB-Lys^60^ (18). Other studies confirmed the ineffective digestion of Lys^48^- and Lys^63^-linked ubiquitin chains by USP7 (19) as well as its poor activity in digesting ubiquitin-ubiquitin linkages or removing Lys^48^-linked ubiquitin chains from autoubiquitinated GST-UbcH5a as a model substrate (15). By contrast, Kategaya *et al.* (20) recently reported that the exo-cleavage activity of USP7 against Lys^48^-linked ubiquitin chains completely removes ubiquitin chains from a polyubiquitinated model peptide. The molecular mechanism by which USP7 removes ubiquitin chains from natural polyubiquitinated substrates remains to be elucidated.

Proliferating cell nuclear antigen (PCNA) is a homotrimer that forms a ring-shaped structure that encircles dsDNA and tethers many DNA metabolic enzymes such as DNA polymerases via PCNA-interacting protein (PIP) boxes (Q*XX*h*XX*aa) or AlkB homologue 2 PCNA-interacting motifs (K/RFhhK/R) (21–23). In response to certain types of DNA damage, monoubiquitinated and Lys^63^-linked polyubiquitinated PCNA at Lys^164^ are accumulated (24–26). Monoubiquitinated and polyubiquitinated PCNA recruits translesion DNA polymerases and ZRANB3 translocase, respectively, to restore the stalled replication at the damage bases (25–32). The recruitment of translesion DNA polymerases is a double-edged sword because of their extremely low fidelity of DNA synthesis, which causes mutagenesis. The tight regulation of translesion DNA polymerases is achieved through the combined action of USP7 and USP1, another deubiquitinase for PCNA, which reverse monoubiquitination and minimize the risk of mutagenesis (33, 34). However, their role in the modulation of polyubiquitinated PCNA remains to be examined.

In the present study, we analyzed the mechanism underlying the deubiquitination of Lys^63^-linked polyubiquitinated PCNA. The results demonstrated that USP7 removes Lys^63^-linked polyubiquitin chains by catalyzing the preferential digestion of PCNA-ubiquitin linkages. USP7 also showed preferential activity against substrate-ubiquitin linkages of the Lys^48^-linked polyubiquitinated p53. This property of USP7 could be beneficial to suppress polyubiquitin signals.

## Results

**Figure 1.**
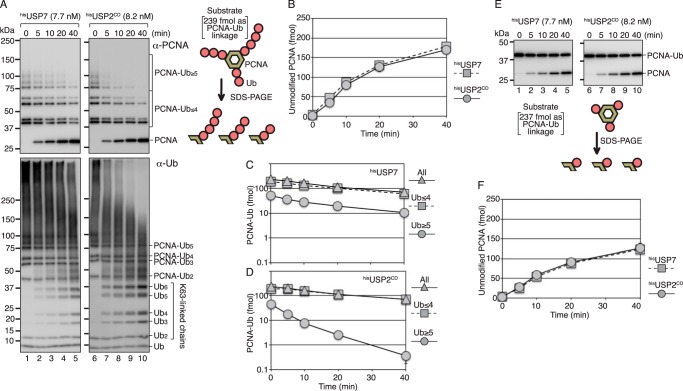
**Isopeptidase activity of USP7 and USP2 for polyubiquitinated and monoubiquitinated PCNA.** *A*, the polyubiquitinated PCNA substrate (80 fmol as a trimer) consisted of 239 fmol of modified PCNA subunits and 1 fmol of unmodified PCNA subunits. The ubiquitinated PCNA contained 195 fmol of PCNA subunits with 1- to 4-mer ubiquitin chains (PCNA-Ub _≤4_), and 44 fmol of PCNA subunits with ubiquitin chains longer than 5 mer (PCNA-Ub _≥5_) (*lanes 1* and *6*). The substrate was incubated with ^his^USP7 (7.7 nm) or ^his^USP2^CD^ (8.2 nm) for the indicated times. The reaction products were analyzed by Western blotting with an anti-PCNA mAb (*upper panels*) or an anti-ubiquitin mAb (*lower panels*). *B–D*, quantified data. Signal intensities of the indicated areas in (*A*) (PCNA, PCNA-Ub _≤4_, and PCNA-Ub _≥5_) were measured, and the relative amounts of PCNA (unmodified), PCNA-Ub _≤4_, PCNA-Ub _≥5_, and total modified PCNA were calculated. The averages of two independent experiments were plotted. In most cases, the *error bars* were smaller than the *symbols*. The amounts of unmodified PCNA generated in the reactions (*B*) and the amounts of remaining ubiquitinated PCNA in the reaction with ^his^USP7 (*C*) or ^his^USP2^CD^ (*D*) are shown. *E*, the monoubiquitinated PCNA substrate (80 fmol as a trimer) consisting of 237 fmol of modified PCNA subunits and 3 fmol of unmodified PCNA subunits was incubated with ^his^USP7 (7.7 nm) or ^his^USP2^CD^ (8.2 nm) for the indicated times. The reaction products were analyzed by Western blotting with an anti-PCNA mAb. *F*, quantified data. Signal intensities of PCNA and PCNA-Ub_1_ in (*E*) were measured, and the relative amounts of PCNA (unmodified) were calculated. The averages of two independent experiments were plotted. The *error bars* were smaller than the *symbols*.

The biochemical activity of USP7 against Lys^63^-linked polyubiquitinated PCNA was analyzed using a mixture of polyubiquitinated PCNA generated *in vitro*. Because Lys^164^ of PCNA is the sole target of ubiquitination, a single Lys^63^-linked ubiquitin chain is attached to each subunit of the PCNA homotrimer (Fig. 1*A*) (35). Most of the polyubiquitinated PCNA monomer had single ubiquitin or ∼7 mer Lys^63^-linked ubiquitin chains as determined by Western blotting with an anti-PCNA mAb (Fig. 1*A*, *upper panels*, *lanes 1* and *6*, and Fig. S1*B*). An anti-ubiquitin mAb (P4D1) detected polyubiquitinated PCNA molecules with long ubiquitin chains as higher-molecular-weight signals (Fig. 1*A*, *lower panels*, *lanes 1* and *6*, and Fig. S1, *A* and *B*), suggesting that a small fraction of PCNA monomer has long ubiquitin chains. Because the anti-ubiquitin antibody detected all ubiquitin molecules in the chains, the PCNA molecules with longer ubiquitin chains were more sensitive to the anti-ubiquitin antibody. We confirmed that the slower migrating molecules were not aggregates of ubiquitinated PCNA, which were detected by treatment with 4 m urea before electrophoresis (Fig. S1*B*).

The monoubiquitinated PCNA substrate (8 nm as a trimer) was subjected to deubiquitination assays with histidine-tagged recombinant USP7 (^his^USP7) (Fig. S2). A histidine-tagged truncated ubiquitin-specific protease 2 (USP2) consisting of the 258–605 amino acid catalytic domain (^his^USP2^CD^) (36) was used as a control for the deubiquitination assay. The time course of the reaction of 7.7 nm ^his^USP7 or 8.2 nm ^his^USP2^CD^ is shown in Fig. 1*A*. Deubiquitinating activity was quantified by measuring the signal intensity from gel images of Western blotting with the anti-PCNA antibody (Fig. 1*A*, *upper panels*). Polyubiquitinated PCNA with short chains (1–4 mer ubiquitin) and long chains (more than 5 mer ubiquitin) relative to that of unmodified PCNA were quantified from gel images (Fig. 1, *B–D*). As shown in Fig. 1*B*, 7.7 nm ^his^USP7 and 8.2 nm ^his^USP2^CD^ generated equal amounts of unmodified PCNA during the 40 min incubation, indicating that the two enzymes have equivalent activities in terms of generating unmodified PCNA from Lys^63^-linked polyubiquitinated PCNA. The catalytic rate for generation of unmodified PCNA was 0.11 (± 0.013) min^−1^ for ^his^USP7 and 0.092 (± 0.026) min^−1^ for ^his^USP2^CD^ under the same reaction conditions (see Fig. 7*A* and Table S1). However, the pattern of reduction of Lys^63^-linked polyubiquitinated PCNA differed between ^his^USP7 and ^his^USP2^CD^. Polyubiquitinated PCNA substrates with shorter and longer ubiquitin chains were similarly reduced in the presence of ^his^USP7 (Fig. 1*C*), whereas polyubiquitinated PCNA substrates with longer ubiquitin chains were rapidly reduced in the presence of ^his^USP2^CD^ (Fig. 1*D*). This result suggested that USP7 preferentially digests the isopeptide linkage between PCNA and ubiquitin rather than the Lys^63^ linkages between ubiquitin molecules. However, this result does not exclude the possibility that USP7 digested Lys^63^-linked ubiquitin chains from the distal end of the chain with extreme processivity. We assumed that the preferential digestion of the PCNA-ubiquitin linkage would generate unanchored Lys^63^-linked ubiquitin chains, whereas digestion of the Lys^63^ linkage from the distal end of the chain would not generate detectable unanchored ubiquitin chains. To clarify the action of USP7, the reaction products were analyzed by Western blotting with the anti-ubiquitin antibody. It is noteworthy that this antibody is poor at detecting monoubiquitinated PCNA and ubiquitin monomers, probably because of its lower affinity for the single ubiquitin moiety and free ubiquitin. The results demonstrated that incubation with ^his^USP7 generated unanchored ubiquitin chains (Fig. 1*A*, *bottom left panel*). This result could exclude the possibility of preferential digestion from the distal end of the chain with extreme processivity. Strikingly, the higher-molecular-weight signals were relatively resistant to ^his^USP7 (Fig. 1*A*, *bottom left panel*), whereas they were more sensitive to ^his^USP2^CD^ (Fig. 1*A*, *bottom right panel*). These results suggested that USP7 preferentially digested PCNA-ubiquitin linkages, but not Lys^63^-linked polyubiquitin chains. In the following experiments, we analyzed the molecular mechanism underlying the preferential digestion of the PCNA-ubiquitin linkages of Lys^63^-linked polyubiquitinated PCNA.

First, we tested the possibility that USP7 has higher activity toward the PCNA-ubiquitin linkage than toward the Lys^63^-linked ubiquitin linkage. To this end, we individually measured isopeptidase activities for the PCNA-ubiquitin linkage and the Lys^63^ linkage between ubiquitin molecules. The digestion of isopeptide linkages between PCNA and ubiquitin was examined using 8 nm monoubiquitinated PCNA trimer as the substrate (Fig. 1, *E* and *F*). The time course of the reaction with 7.7 nm ^his^USP7 or 8.2 nm ^his^USP2^CD^ demonstrated that both enzymes released the same amount of unmodified PCNA from monoubiquitinated PCNA (Fig. 1*F*). The catalytic rate for the generation of unmodified PCNA was 0.068 (± 0.0059) min^−1^ for ^his^USP7 and 0.067 (± 0.0067) min^−1^ for ^his^USP2^CD^ under the same reaction conditions (see Fig. 7*A* and Table S1).

**Figure 2.**
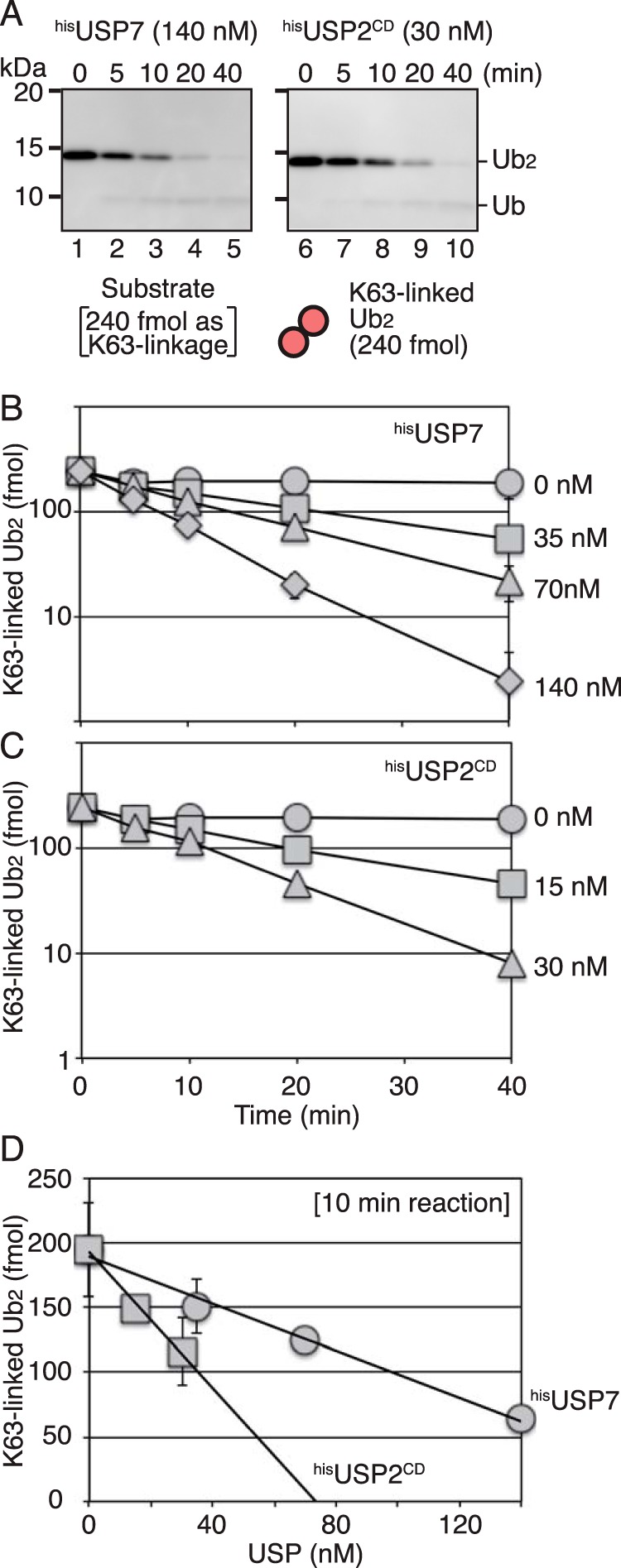
**Isopeptidase activity of USP7 and USP2 for Lys^63^-linked diubiquitin.** *A*, Lys^63^-linked diubiquitin substrate of 240 fmol was incubated with ^his^USP7 (140 nm) or ^his^USP2^CD^ (30 nm) for the indicated times. The reaction products were analyzed by Western blotting with an anti-ubiquitin mAb. *B* and *C*, quantified data. The reactions were performed with the indicated concentration of ^his^USP7 (*B*) or ^his^USP2^CD^ (*C*). Signal intensities of diubiquitin were measured, and the relative amount of diubiquitin was normalized to that at time 0. The averages of two independent experiments were plotted. In most cases, the *error bars* were smaller than the *symbols. D*, the data obtained at 10 min in the time course experiments described in (*B*) and (*C*) were replotted against enzyme concentrations.

Second, the isopeptidase activity against the Lys^63^-linkage was examined using 24 nm Lys^63^-linked diubiquitin as the substrate. The time course reactions were performed with different concentrations of ^his^USP7 (Fig. 2*B*) or ^his^USP2^CD^ (Fig. 2*C*). Representative gel images are shown in Fig. 2*A*. Because the anti-ubiquitin antibody used in this study detects ubiquitin monomers and diubiquitin chains with different efficacy (Fig. 2*A*), the deubiquitination activity was quantified by determining the amount of diubiquitin relative to that at time 0 (Fig. 2, *B* and *C*). The data at 10 min in the reaction with different amounts of ^his^USP7 or ^his^USP2^CD^ obtained from the time course (Fig. 2, *B* and *C*) were replotted against enzyme concentration (Fig. 2*D*). At 10 min in the absence of enzyme, the substrate was reduced to ∼80% (∼200 fmol) of the initial amount at time 0 (Fig. 2*D*). The formation of aggregates or absorption to the tube walls may account for ∼20% of the lost substrate. The catalytic rate for digestion of Lys^63^-linked diubiquitin was 0.011 (± 0.0018) min^−1^ for ^his^USP7 and 0.031 (± 0.0030) min^−1^ for ^his^USP2^CD^ under the same reaction conditions (see Fig. 7, *A* and *B*, and Table S1), which was 6.2- and 2.2-fold lower than that measured for the digestion of the PCNA-ubiquitin linkage of monoubiquitinated PCNA, respectively. These results were consistent with the results of that USP7, but not USP2^CD^, preferentially digested the PCNA-ubiquitin linkage of the Lys^63^-linked polyubiquitinated PCNA.

**Figure 3.**
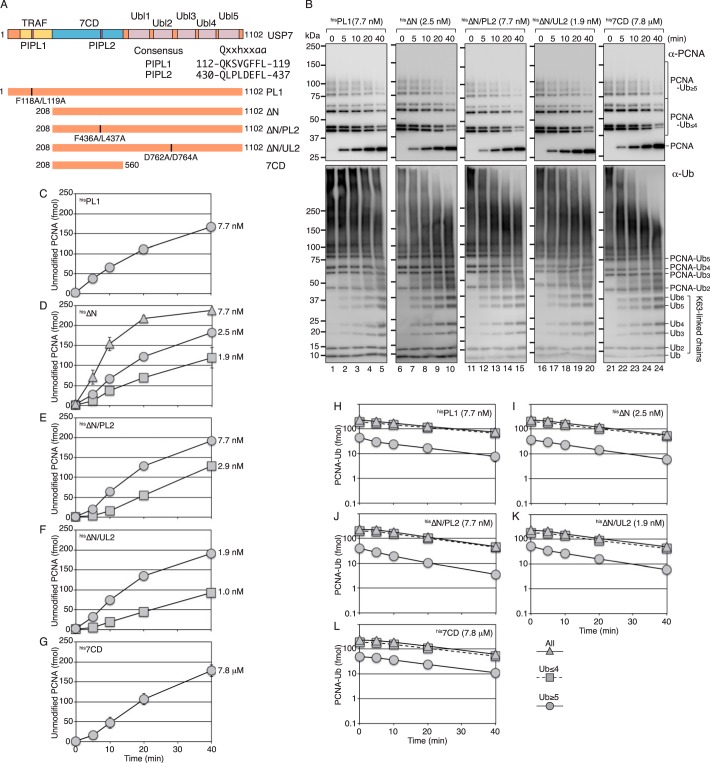
**Isopeptidase activity of USP7 mutants for polyubiquitinated PCNA.** *A*, schematic representation of the structure of USP7 and its mutants. An alignment of PIPL1 and PIPL2 with the PIP-consensus sequence is shown. *B*, the reactions were performed with the indicated mutants and analyzed as described in Fig. 1*A. C–L*, quantified data of isopeptidase activity for polyubiquitinated PCNA. The relative amounts of reaction products were calculated as described in Fig. 1*B*. The amounts of unmodified PCNA generated in the reactions with the indicated concentrations of ^his^PL1 (*C*), ^his^ΔN (*D*), ^his^ΔN/PL2 (*E*), ^his^ΔN/UL2 (*F*), or ^his^7CD (*G*) and the amounts of remaining ubiquitinated PCNA in the reactions with the indicated concentrations of ^his^PL1 (*H*), ^his^ΔN (*I*), ^his^ΔN/PL2 (*J*), ^his^ΔN/UL2 (*K*), or ^his^7CD (*L*) are shown. The averages of at least two independent experiments were plotted. In most cases, the *error bars* were smaller than the *symbols*.

To determine whether the interaction between USP7 and PCNA is required for the preferential digestion of the PCNA-ubiquitin linkage, we first investigated the direct interaction between USP7 and PCNA in *in vitro* binding assays. However, no interaction was detected (Fig. S3). Nonetheless, low-affinity or undetectable binding mediated by the substrate-binding domains, N-terminal TRAF or Ubl2, may play a role in the preferential digestion of the PCNA-ubiquitin linkage. Regarding the interaction with PCNA, a PIP boxlike sequence (PIPL1) in the TRAF domain and a second PIP boxlike sequence (PIPL2) in the USP7 catalytic domain (7CD) (Fig. 3*A*) were detected and considered as candidates for the low-affinity interaction with PCNA. To examine whether these sequences are involved in the preferential digestion of the PCNA-ubiquitin linkage, we generated a PIPL1 mutant F118A/L119A (designated ^his^PL1), a TRAF-truncated mutant (designated ^his^ΔN), a construct with an additional mutation in PIPL2 (F436A/L437A, designated ^his^ΔN/PL2) or in the target protein-binding motif of Ubl2 (12) (D762A/D764A, designated ^his^ΔN/UL2), and a truncated mutant consisting of the 7CD (6) (designated ^his^7CD) (Fig. 3*A* and Fig. S2). The time courses of the deubiquitination reactions of polyubiquitinated PCNA with 7.7 nm ^his^PL1, 2.5 nm ^his^ΔN, 7.7 nm ^his^ΔN/PL2, 1.9 nm ^his^ΔN/UL2, and 7.8 μm ^his^7CD are shown in Fig. 3*B* (*upper panels*) and Fig. 3, *C–G*. The results showed that almost equivalent amounts of unmodified PCNA were released in the presence of the indicated concentrations of the mutants. Because of the low activity of ^his^7CD (6, 10) (∼1000-fold lower than that of ^his^USP7), it was not subjected to further quantitative analysis. The catalytic rate for the generation of unmodified PCNA was 0.092 (±0.0065) min^−1^ for ^his^PL1, 0.25 (± 0.027) min^−1^ for ^his^ΔN, 0.086 (± 0.0039) min^−1^ for ^his^ΔN/PL2, and 0.36 (± 0.025) min^−1^ for ^his^ΔN/UL2 (see Fig. 7*A* and Table S1). The pattern of reduction of Lys^63^-linked polyubiquitinated PCNA in the mutants was similar to that in WT ^his^USP7 (Fig. 3, *H–L*). Polyubiquitinated PCNA substrates with shorter and longer ubiquitin chains were similarly reduced in the reactions with all mutants (Fig. 3, *H–L*). These results showed that the preferential digestion of the isopeptide linkage between PCNA and ubiquitin was retained in all mutants, suggesting that this property is not a consequence of the specific interaction with PCNA, but rather intrinsic to the 7CD. However, when the same products were analyzed by Western blotting with the anti-ubiquitin antibody, significant digestion of ubiquitin chains was detected in the ΔN, ΔN/PL2, and 7CD and less efficiently in ΔN/UL2, but not in PL1 (Fig. 3*B*, *lower panels*). These results were inconsistent with those detected with the anti-PCNA antibody (Fig. 3*B*, *upper panels*). One possible interpretation is that the TRAF-truncated mutants gained the ability to digest long ubiquitin chains, but not relatively short ubiquitin chains of ∼7 mer detectable with the anti-PCNA antibody (Fig. 3*B*, *upper panels*) (see below).

**Figure 4.**
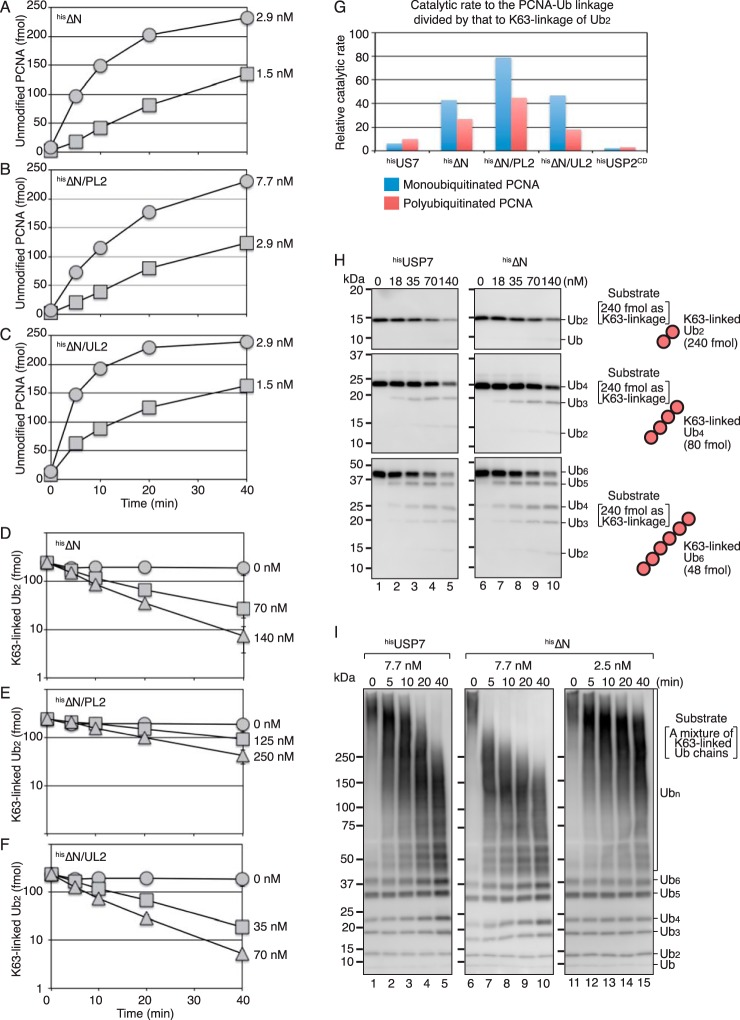
**Isopeptidase activity of USP7 mutants for monoubiquitinated PCNA and ubiquitin chains.** *A–C*, isopeptidase activity for monoubiquitinated PCNA. The reactions were performed as described in Fig. 1*E*. The amounts of unmodified PCNA generated in the reactions with the indicated concentrations of ^his^ΔN (*A*), ^his^ΔN/PL2 (*B*), or ^his^ΔN/UL2 (*C*) are shown. *D–F*, isopeptidase activity for Lys^63^-linked diubiquitin. The reactions were performed as described in Fig. 2*A*. The amounts of the remaining Lys^63^-linked diubiquitin in the reactions with the indicated concentrations of ^his^ΔN (*D*), ^his^ΔN/PL2 (*E*), or ^his^ΔN/UL2 (*F*) are shown. The averages of two independent experiments were plotted. In most cases, the *error bars* were smaller than the *symbols. G*, preferential cleavage of the PCNA-ubiquitin linkage of monoubiquitinated or polyubiquitinated PCNA over the Lys^63^-diubiquitin linkage. The catalytic rate for the PCNA-ubiquitin linkage was divided by that for Lys^63^-linked diubiquitin. *H*, Lys^63^-linked diubiquitin (*top panels*), tetra-ubiquitin (*middle panels*), or hexa-ubiquitin (*bottom panels*) (240 fmol) as Lys^63^ linkage was incubated with the indicated concentrations of ^his^USP7 or ^his^ΔN for 10 min. The reaction products were analyzed by Western blotting with an anti-ubiquitin mAb. *I*, a mixture of Lys^63^-linked polyubiquitin chains was incubated with the indicated concentration of ^his^USP7 or ^his^ΔN for the indicated times. The reaction products were analyzed by Western blotting with an anti-ubiquitin mAb.

To further elucidate the biochemical properties of the ^his^ΔN mutants, we determined their catalytic rates for the respective linkages. The time courses of the reactions with monoubiquitinated PCNA as substrate are shown in Fig. 4, *A–C*. The catalytic rate for the generation of unmodified PCNA from monoubiquitinated PCNA was 0.40 (± 0.16) min^−1^ for ^his^ΔN, 0.15 (± 0.030) min^−1^ for ^his^ΔN/PL2, and 0.94 (± 0.10) min^−1^ for ^his^ΔN/UL2 (see Fig. 7*A* and Table S1). The time courses of the reactions with Lys^63^-linked diubiquitin as a substrate are shown in Fig. 4, *D–F*. The catalytic rate for digestion of the Lys^63^ linkage between ubiquitin molecules was 0.0093 (± 0.0018) min^−1^ for ^his^ΔN, 0.0019 (± 0.00014) min^−1^ for ^his^ΔN/PL2, and 0.020 (± 0.0018) min^−1^ for ^his^ΔN/UL2 under the same reaction conditions (see Fig. 7, *A* and *B*, and Table S1). All the ^his^ΔN mutants showed increased preference for the isopeptide linkages between PCNA and ubiquitin in both monoubiquitinated and polyubiquitinated PCNA over that of Lys^63^-linked diubiquitin (Fig. 4*G*). This was consistent with the finding that the TRAF-truncated mutants as well as full-length USP7 preferentially digested the PCNA-ubiquitin linkages of the Lys^63^-linked polyubiquitinated PCNA with relatively short ubiquitin chains (∼7 mer). To investigate the effect of chain length, we tested Lys^63^-linked tetra- and hexa-ubiquitin and a mixture ranging from dimer to very long ubiquitin chains (Fig. S1). As shown in Fig. 4*H*, Lys^63^-linked tetra-, and hexa-ubiquitin were digested at a similar rate as diubiquitin by both ^his^USP7 and ^his^ΔN. By contrast, a clear difference between ^his^USP7 and ^his^ΔN was observed in the presence of the mixture from dimer to very long ubiquitin chains. The long ubiquitin chains were sensitive to ^his^ΔN (Fig. 4*I*, *lanes 6–10*), whereas they were relatively resistant to ^his^USP7 (Fig. 4*I*, *lanes 1–5*). The rapid digestion of the long chains observed with 7.7 nm ^his^ΔN did not appear to be a consequence of a processive reaction, because the chain lengths were uniformly increased under reduced concentrations of ^his^ΔN (2.5 nm) (Fig. 4*I*, *lanes 11–15*). These results suggested that the TRAF domain negatively regulates the isopeptidase activity of USP7 toward Lys^63^-linked long ubiquitin chains; therefore, the TRAF domain could enhance the preference for the PCNA-ubiquitin linkages of the polyubiquitinated PCNA with long chains.

**Figure 5.**
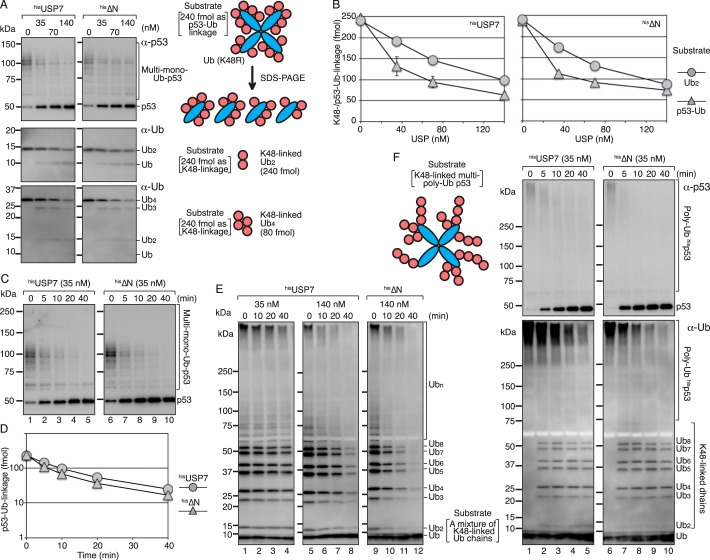
**Isopeptidase activity of USP7 for ubiquitinated p53 and Lys^48^-linked ubiquitin chains.** *A*, the multi-monoubiquitinated p53 substrate (240 fmol) as p53-ubiquitin linkage (*top panels*), or Lys^48^-linked diubiquitin (*middle panels*), or tetra-ubiquitin (*bottom panels*) (240 fmol) as Lys^48^ linkage was incubated with the indicated concentration of ^his^USP7 or ^his^ΔN for 10 min. The reaction products were analyzed by Western blotting with the indicated antibody. *B*, quantified data. For the p53-ubiquitin linkage, the amounts of linkages remaining after the incubation period were calculated as described in Fig. S4. For the Lys^48^-linked diubiquitin, signal intensities of diubiquitin were measured, and the relative amounts of diubiquitin were normalized to that at time 0. The averages of two independent experiments were plotted. In most cases, the *error bars* were smaller than the *symbols. C* and *D*, time courses of the reactions. The reactions were performed with the multi-monoubiquitinated p53 substrate (240 fmol) as p53-ubiquitin linkage and the indicated concentration of enzymes (*C*) and quantified (*D*) as described in (*B*). The averages of two independent experiments were plotted. The *error bars* were smaller than the *symbols. E*, a mixture of Lys^48^-linked polyubiquitin chains was incubated with the indicated concentration of ^his^USP7 or ^his^ΔN for the indicated times. The reaction products were analyzed by Western blotting with an anti-ubiquitin mAb. *F*, the multi-polyubiquitinated p53 substrate was incubated with 35 nm ^his^USP7 or ^his^ΔN for the indicated times. The reaction products were analyzed by Western blotting with the indicated antibodies.

Next, we asked whether the preference of USP7 for the substrate-ubiquitin linkage of the polyubiquitinated substrate is specific to PCNA. As another substrate, p53-ubiquitin linkages were examined. To determine the isopeptidase activity toward p53-ubiquitin linkages, a mixture of multi-monoubiquitinated p53 substrate was generated *in vitro* with the ^his^E6AP-E6-p53 complex and K48R mutant ubiquitin (Fig. S1*A*). Under ubiquitination conditions, more than 95% of ubiquitin molecules were singly attached to p53.^3^ The average number of p53-ubiquitin linkages in the mixture of multi-monoubiquitinated p53 substrates was estimated at 5.1 per p53 subunit (Fig. S4). An amount corresponding to 240 fmol of p53-ubiquitin linkages was subjected to deubiquitination assays (Fig. 5, *A*, *top panels*, and *B–D*). The isopeptidase activity for the Lys^48^ linkage between ubiquitin molecules was also examined using Lys^48^-linked diubiquitin (Fig. 5, *A*, *middle panels,* and *B*). The catalytic rate for digestion of p53-ubiquitin linkages was 0.042 (± 0.0096) and 0.054 (± 0.0016) min^−1^ for ^his^USP7 and ^his^ΔN, respectively, and that for the Lys^48^ linkage of diubiquitin was 0.016 (±0.0010) and 0.019 (±0.0035) min^−1^, respectively (see Fig. 7, *A* and *B*, and Table S1). These results indicated that Lys^48^-linked diubiquitin was a poor substrate as well as Lys^63^-linked diubiquitin, and p53-ubiquitin linkages were better substrates for both ^his^USP7 and ^his^ΔN. We also tested Lys^48^-linked tetra-ubiquitin (Fig. 5*A*, *bottom panels*) and a mixture of Lys^48^-linked ubiquitin chains ranging from dimer to more than octamer (Fig. 5*E*, *lanes 5–12*). The results showed that both ^his^USP7 and ^his^ΔN similarly digested those chains. The digestion of multi-monoubiquitinated p53 with 35 nm ^his^USP7 or ^his^ΔN was more efficient than that of the mixture of Lys^48^-linked ubiquitin chains with 35 nm ^his^USP7 (compare Fig. 5, *C* and *E*, *lanes 1–4*), confirming that p53-ubiquitin linkages are a better substrate than Lys^48^-linked ubiquitin-ubiquitin linkages. Furthermore, the preferential digestion of the p53-ubiquitin linkage of polyubiquitinated p53 was analyzed using multi-polyubiquitinated p53 as a substrate (Fig. S1*A*). In the presence of 35 nm of ^his^USP7 or ^his^ΔN, unmodified p53 was generated from multi-polyubiquitinated p53 (Fig. 5*F*, *upper panels*) as well as from multi-monoubiquitinated p53 (Fig. 5*C*). By contrast, the released Lys^48^-linked ubiquitin chains were relatively resistant (Fig. 5*F*, *lower panels*). This was consistent with the observation that Lys^48^-linked ubiquitin chains themselves were largely resistant to 35 nm ^his^USP7 (Fig. 5*E*, *lanes 1–4*).

**Figure 6.**
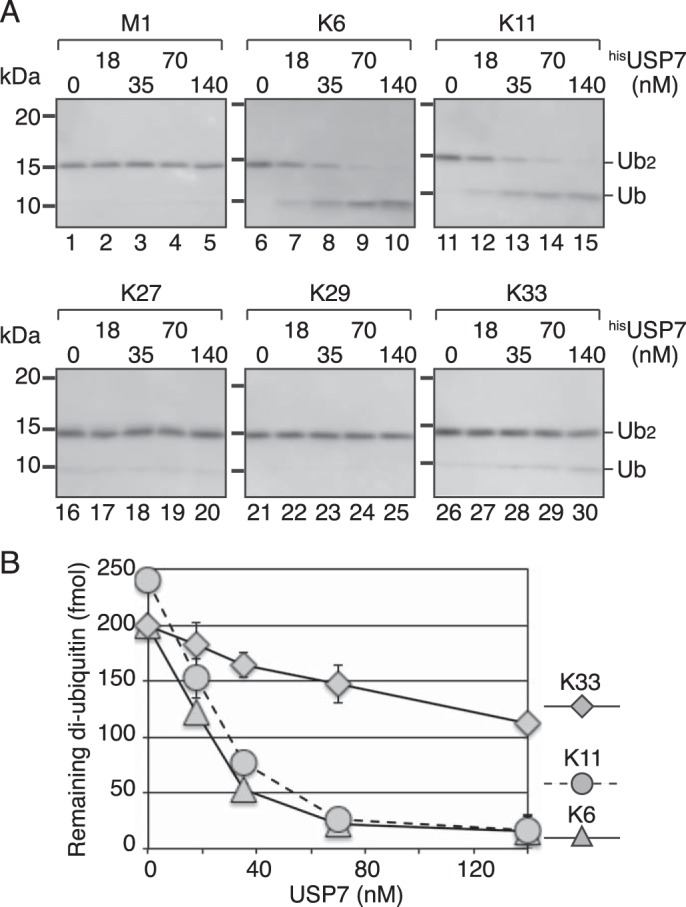
**Isopeptidase activity of USP7 for diubiquitin with various linkages.** *A*, each diubiquitin with the indicated linkage (240 fmol) was incubated with the indicated concentration of ^his^USP7 for 10 min. The reaction products were analyzed by Western blotting with an anti-ubiquitin mAb. *B*, quantified data. Signal intensities of diubiquitin in (*A*) were measured, and the relative amount of diubiquitin was normalized to that at time 0. In the cases of Lys^6^- and Lys^33^-linked diubiquitin, the substrates were reduced to ∼80% of the initial amount during a 10-min incubation without enzymes. The averages of two independent experiments were plotted. In most cases, the *error bars* were smaller than the *symbols*.

**Figure 7.**
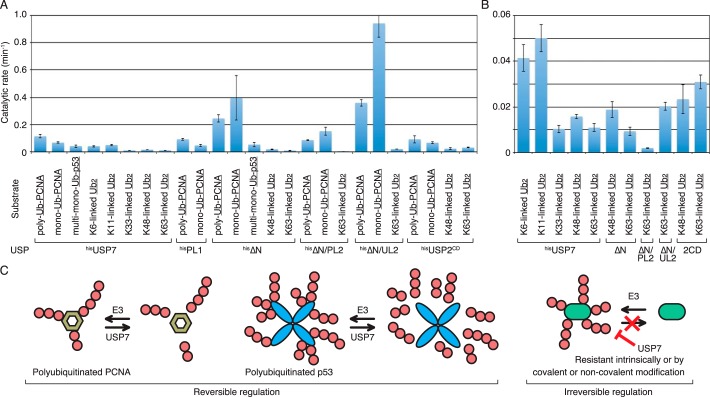
**Summary of isopeptidase activities.** *A*, the rates of the reactions in the presence of substrates containing 24 nm underlined linkages were calculated using 4–16 independent values. Data falling within a linear range in the presence of different amounts of enzymes and time points were adopted. The total amount of digested linkage (fmol) in 10 μl of the reaction mixture was divided by the amounts of enzymes (fmol) and reaction time (min). *B*, the same data of the rates of the reactions with diubiquitin are shown in a different scale. *2CD* represents ^his^USP2^CD^. *C*, schematic representation of USP7 function. Natural substrates for USP7 are regulated by reversible ubiquitination (*left*). Some proteins, which are intrinsically or modification-dependently resistant to substrate-ubiquitin linkage-specific deubiquitinases such as USP7, might be irreversibly regulated (*right*).

Lastly, we examined whether the poor activity toward Lys^48^- and Lys^63^-linked diubiquitin was a property of the diubiquitin linkage. As shown in Fig. 6, ^his^USP7 hardly digested Met^1^-, Lys^27^-, and Lys^29^-linked diubiquitin. By contrast, Lys^6^- and Lys^11^-linked diubiquitin were good substrates. Lys^33^-linked diubiquitin was digested at a level similar to Lys^48^- and Lys^63^-linked diubiquitin (Figs. 6 and 7*B* and Table S1). These results indicated that the isopeptidase activity of USP7 toward diubiquitin varied depending on the type of isopeptide linkage.

## Discussion

In the present study, the catalytic rate of USP7 toward various natural substrates under the same assay conditions was analyzed for the first time, and the mechanism underlying the deubiquitination of polyubiquitinated substrates by USP7 was elucidated. Because obtaining highly concentrated natural substrates to perform standard Michaelis-Menten kinetic experiments was difficult, we examined the catalytic rates under a constant substrate concentration of 24 nm. The results demonstrated that USP7 preferentially digested the substrate-ubiquitin linkages of the two natural substrates, Lys^63^-linked polyubiquitinated PCNA and Lys^48^-linked multi-polyubiquitinated p53. This property was attributed to the individual catalytic rate toward the respective linkages of PCNA-ubiquitin, p53-ubiquitin, and ubiquitin-ubiquitin. Because USP7 has weak activity toward Lys^48^- and Lys^63^-linked ubiquitin chains, the reaction is not an absolute *en bloc* deubiquitinating reaction. These results are consistent with those of previous reports (15, 18, 19). Kategaya *et al.* (20) recently proposed that USP7 efficiently removes ubiquitin chains from a polyubiquitinated model peptide through its exo-cleavage activity toward Lys^48^-linked ubiquitin chains. This result does not exclude the possibility that the unmodified peptide was released by preferential digestion of the substrate-ubiquitin linkage over the exo-cleavage activity, as the proportion of ubiquitin monomers and ubiquitin chains generated under the reaction was not examined (20).

To compare the results with previously published data, the catalytic rate in the presence of 24 nm substrate was calculated based on the Michaelis-Menten equation as follows: *v* = (*V*_max_ · [S])/(*K_m_* + [S]). Thus, *k*_cat_ was used to determine the rate (min^−1^) in the presence of 25 nm substrate using the following equation: (*k*_cat_ · [25 nm])/(*K_m_* + [25 nm]), where *k*_cat_ and *K_m_* are previously reported parameters (Table S1). The catalytic rates of USP7 and USP2^CD^ for 24 nm PCNA-ubiquitin linkage of polyubiquitinated PCNA were 2- to 6-fold lower than those for 24 nm Ub-AMC calculated according to the reported parameters (Table S1) (6, 10, 20, 36, 37, 38, 42). The calculated rates in the presence of 24 nm Lys^48^- and Lys^63^-linked diubiquitin from published parameters (37) are 10- and 8-fold higher than the catalytic rates determined in the present study (Table S1). We confirmed that the poor catalytic activity observed in the present study was not related to the buffer conditions (data not shown). The catalytic rate of USP2^CD^ for Lys^48^-linked diubiquitin determined in the present study was also 4.4-fold lower than that calculated according to published parameters (Table S1) (38). Previous studies determined the kinetic parameters using standard steady-state kinetics experiments in the presence of high concentrations of substrates (37, 38). The discrepancy between experiments using low and high concentrations of substrate remains to be clarified.

We demonstrated that the levels of isopeptidase activity of USP7 varied depending on the type of isopeptide linkage. The PCNA-ubiquitin linkage was the most efficiently digested. The p53-ubiquitin linkage and the ubiquitin linkages of Lys^6^- and Lys^11^-linked diubiquitin were digested similarly. The ubiquitin linkages of Lys^33^-, Lys^48^-, and Lys^63^-linked diubiquitin were poor substrates, and those of Lys^27^- and Lys^29^-linked diubiquitin as well as the liner diubiquitin were hardly digested. The trend in the catalytic activity toward the respective linkage of diubiquitin was consistent with that reported previously (37). These results suggested that the surface structure in which the amine forms an isopeptide bond with the C-terminal of ubiquitin was involved in the efficiency of cleavage. Previous reports indicated that the substrate-ubiquitin linkages of model substrates were poorly digested by USP7. Canning *et al.* (15) showed that USP7 does not efficiently digest UBCH5a-ubiquitin linkages. Schaefer and Morgan (18) demonstrated that USP7 is ineffective at removing Lys^48^-linked ubiquitin chains from polyubiquitinated CycB-Lys^60^, whereas it efficiently digests the substrate-ubiquitin linkage of monoubiquitinated CycB-Lys^60^. By contrast, the p53-ubiquitin linkages of monoubiquitinated and polyubiquitinated p53 are similarly digested by USP7 (10, 15–17). We suggest that the configurations of the ubiquitin chains attached to CycB-Lys^60^ and the ubiquitin molecules attached to UBCH5a are not suitable for digestion by USP7, thereby preventing deubiquitination.

We could postulate that there are two types of polyubiquitinated proteins: One type can be deubiquitinated to enable reversible regulation through the balance between ubiquitination and deubiquitination. Substrates of USP7 such as PCNA and p53 may belong to this group of proteins (Fig. 7*C*). Here, preferential digestion of the substrate-ubiquitin linkage results in the rapid and effective suppression of polyubiquitin signals. In the other substrates, the substrate-ubiquitin linkage, such as the cyclin B–ubiquitin linkage, is not digested by USPs with properties similar to those of USP7. In addition to the intrinsically inaccessible substrate-ubiquitin linkages, covalent modifications such as phosphorylation or binding of proteins or small molecules may prevent the cleavage of substrate-ubiquitin linkages by specific USPs. We suggest that such mechanism for preventing deubiquitination through the sequestration of substrate-ubiquitin linkages might regulate the fate of polyubiquitinated substrates of USP7 and of other substrate-ubiquitin linkage-specific USPs.

## Experimental procedures

### Western blotting

Anti-E6AP anti-serum was raised in a rabbit against the peptide KKGPRVDPLETELGVKTLDC. Antibodies used included anti-PCNA (Santa Cruz Biotechnology, PC10, sc-56), anti-p53 (Calbiochem, Ab-6 mouse mAb (DO-1), OP43), anti-UBCH5c (Ab Frontier (4C1-1E3), LF-MA10362), anti-USP7 (Abcam, ab4080), and anti-ubiquitin (Santa Cruz Biotechnology, P4D1, sc-8017). Signals were detected with a Chemi-Lumi One L kit (Nacalai Tesque, 07880-70) using ImageQuant™ LAS4000 Mini Biomolecular Imager (GE Healthcare), and analyzed using ImageJ 1.48v software (National Institutes of Health).

### Proteins

Recombinant human proteins were obtained from the following sources: K48R mutant ubiquitin (UM-K48R); Lys^6^- (UC-11B), Lys^11^- (UC-40B), Lys^27^- (UC-61B), Lys^29^- (UC-81B), Lys^33^- (UC-101B), Lys^48^- (UC-200B), Lys^63^- (UC-300B), and Met^1^-linked (UC-700B) diubiquitins; Lys^48^- (UC-210B) and Lys^63^-linked (UC-310) tetra-ubiquitins; and Lys^63^-linked hexa-ubiquitin (UC-317) were purchased from Boston Biochem (Cambridge, MA). PCNA, RAD6-(RAD18)_2_ (39, 40), HLTF, MMS2-UBC13, E1, ubiquitin, and ^his^USP2^CD^ (the catalytic domain consisting of 258–605 amino acid residues) (36) were purified as described previously (31, 35, 41). The ^his^E6AP-E6 complex, ^his^p53, ^his^E6AP-E6-p53 complex, UBCH5c, and ^his^UBCH5c were purified as described elsewhere.^3^

Human *USP7* was cloned into the pET-20b(+) vector with the histidine tag sequence of pET-15b to yield pET20-hisUSP7. The histidine tag was designed to be attached at the N terminus of the proteins. Recombinant ^his^USP7 was produced in *Escherichia coli* BL21 (DE3) harboring pET20-hisUSP7 at 15 °C by addition of isopropyl β-d-1-thiogalactopyranoside (0.2 mm), and purified by chromatography at 4 °C using Ni^2+^-charged HiTrap Chelating HP, HiTrap Q HP, and Superdex 200 columns (GE Healthcare). Mutant proteins were purified as described for the WT proteins.

### Substrate preparation

Monoubiquitinated PCNA was prepared as described previously (32). Polyubiquitinated PCNA was prepared as follows: a reaction mixture (1 ml) containing 20 mm HEPES-NaOH, pH 7.5, 50 mm NaCl, 0.2 mg/ml BSA, 1 mm DTT, 5 mm MgCl_2_, 1 mm ATP, poly(dA)-oligo(dT) (GE Healthcare) (4 μg), PCNA (40 pmol as a trimer), RFC (28 pmol), E1 (34 pmol), RAD6A-(RAD18)_2_ complex (22 pmol as a trimer), MMS2-UBC13 complex (40 pmol as a dimer), HLTF (6.6 pmol), and ubiquitin (7 nmol) was incubated at 30 °C for 160 min, and chilled on ice. Aliquots of 20 μl were frozen in liquid N_2_ and stored −80 °C.

A mixture of Lys^63^-linked polyubiquitin chains was prepared as follows: a reaction mixture (1 ml) containing 20 mm HEPES-NaOH, pH 7.5, 50 mm NaCl, 0.2 mg/ml BSA, 1 mm DTT, 5 mm MgCl_2_, 1 mm ATP, poly(dA)-oligo(dT) (GE Healthcare) (4 μg), MMS2-UBC13 complex (650 pmol as a dimer), HLTF (180 pmol), and ubiquitin (7 nmol) was incubated at 30 °C for 40 min, and chilled on ice. Aliquots of 20 μl were frozen in liquid N_2_ and stored −80 °C.

A mixture of Lys^48^-linked polyubiquitin chains was prepared by digestion of poly-ubiquitinated ^his^p53 with ^his^USP7 and subsequent separation of Lys^48^-linked polyubiquitin chains from the reaction mixture as follows. A reaction mixture (1 ml) containing 20 mm HEPES-NaOH, pH 7.5, 50 mm NaCl, 0.02 mg/ml BSA, 1 mm DTT, 5 mm MgCl_2_, 1 mm ATP, ^his^p53 (40 pmol as a tetramer), E1 (34 pmol), ^his^UBCH5c (1.6 nmol), ^his^E6AP-E6 (32 pmol), and ubiquitin (7 nmol) was prepared on ice and then incubated at 30 °C for 20 min. The reaction was terminated by addition of DTT at 5 mm and EDTA at 20 mm, and then incubated with ^his^USP7 at the concentration of 80 nm at 30 °C for 90 min and chilled on ice for 10 min. After addition of imidazole to 24 mm, the reaction mixture was loaded onto Ni^2+^-charged HiTrap Chelating HP and a flow-through fraction was collected. The samples before loading onto the column and the flow-through fraction were analyzed by Western blotting to confirm that ^his^USP7, ^his^p53, ^his^E6AP, and ^his^UBCH5c were eliminated (Fig. S1*C*).

Multi-monoubiquitinated p53 was prepared as follows: a reaction mixture (250 μl) containing 20 mm HEPES-NaOH, pH 7.5, 50 mm NaCl, 0.02 mg/ml BSA, 1 mm DTT, 5 mm MgCl_2_, 1 mm ATP, ^his^E6AP-E6-p53 complex (3.4 pmol as p53 monomer), E1 (8.5 pmol), UBCH5c (12.5 pmol), and K48R mutant ubiquitin (1740 pmol) was prepared. The mixture without ^his^E6AP-E6-p53 complex was incubated at 30 °C for 1 min, and then the ^his^E6AP-E6-p53 complex was introduced into the mixture. After an additional 5 min of incubation, the mixture was chilled on ice. Aliquots of 20 μl were frozen in liquid N_2_ and stored −80 °C.

Multi-polyubiquitinated p53 was prepared as described for multi-polyubiquitinated p53 except for the use of WT ubiquitin and 20 min of incubation for the reaction. After the reaction, EDTA and DTT were introduced at concentrations of 20 mm and 5 mm, respectively. Aliquots of 20 μl were frozen in liquid N_2_ and stored at −80 °C.

### Deubiquitination assays

Deubiquitinating reactions were performed in a reaction mixture (10 μl) containing 20 mm HEPES-NaOH, pH 7.5, 60 mm NaCl, 0.2 mg/ml BSA, 5 mm DTT, 5 mm EDTA, and the indicated substrate at 30 °C for the indicated times with the indicated amounts of ^his^USP7 or its mutants. The reactions were terminated by the addition of SDS sample buffer. Products were analyzed by Western blotting with the indicated antibodies.

## Author contributions

Y. M. conceptualization; Y. M. formal analysis; Y. M., R. K., and C. M. funding acquisition; Y. M. investigation; Y. M. visualization; Y. M. methodology; Y. M. writing-original draft; Y. M. project administration; R. K. validation; R. K., H. K., I. K., and C. M. writing-review and editing; H. K. and I. K. resources; C. M. supervision.

## Supplementary Material

Supporting Information

## Footnotes

3 Y. Masuda, Y. Saeki, N. Arai, H. Kawai, I. Kukimoto, K. Tanaka, and C. Masutani, manuscript in preparation.

## Footnotes

2 The abbreviations used are:

TRAF: tumor necrosis factor receptor-associated factor-like
PCNA: proliferating cell nuclear antigen
Ubl: ubiquitin-like
PIP: PCNA-interacting protein.
