# Supplementary material for: Preferential digestion of PCNA-ubiquitin and p53-ubiquitin linkages by USP7 to remove polyubiquitin chains from substrates
Source: J Biol Chem. 2019 Jan 15;294(11):4177–87. doi: 10.1074/jbc.RA118.005167 (PMC6422070; doi:10.1074/jbc.RA118.005167)
Supplement: Supporting Information [file supp_294_11_4177__index.html]

Preferential digestion of PCNA-ubiquitin and p53-ubiquitin linkages by USP7 to remove polyubiquitin chains from substrates — Isopeptide linkage selectivity of USP7 — Preferential digestion of PCNA-ubiquitin and p53-ubiquitin linkages by USP7 to remove polyubiquitin chains from substrates — Isopeptide linkage selectivity of USP7 — Supporting Information 

# Preferential digestion of PCNA-ubiquitin and p53-ubiquitin linkages by USP7 to remove polyubiquitin chains from substrates

## Supporting Information

- Supporting Information (to be published online) - Fig. S1-S4 and Table S1
