# Supplementary material for: Preferential digestion of PCNA-ubiquitin and p53-ubiquitin linkages by USP7 to remove polyubiquitin chains from substrates
Source: J Biol Chem. 2019 Jan 15;294(11):4177–87. doi: 10.1074/jbc.RA118.005167 (PMC6422070; doi:10.1074/jbc.RA118.005167)
Supplement: Supporting Information [file supp_RA118.005167_139846_1_supp_256570_pk4vv8.pdf]

Preferential digestion of PCNA-ubiquitin and p53-ubiquitin linkages by USP7 to remove polyubiquitin chains from substrates

Yuji Masuda*, Rie Kanao, Hidehiko Kawai, Iwao Kukimoto, and Chikahide Masutani

Supporting information includes the following:

Figure S1. *In vitro*-generated substrates in this study.

Figure S2. Purified proteins.

Figure S3. Pull-down assay of PCNA.

Figure S4. Estimation of the ubiquitin-p53 linkages in the multi-monoubiquitinated p53 substrate.

Table S1. Comparison with published data on the catalytic activity of USP

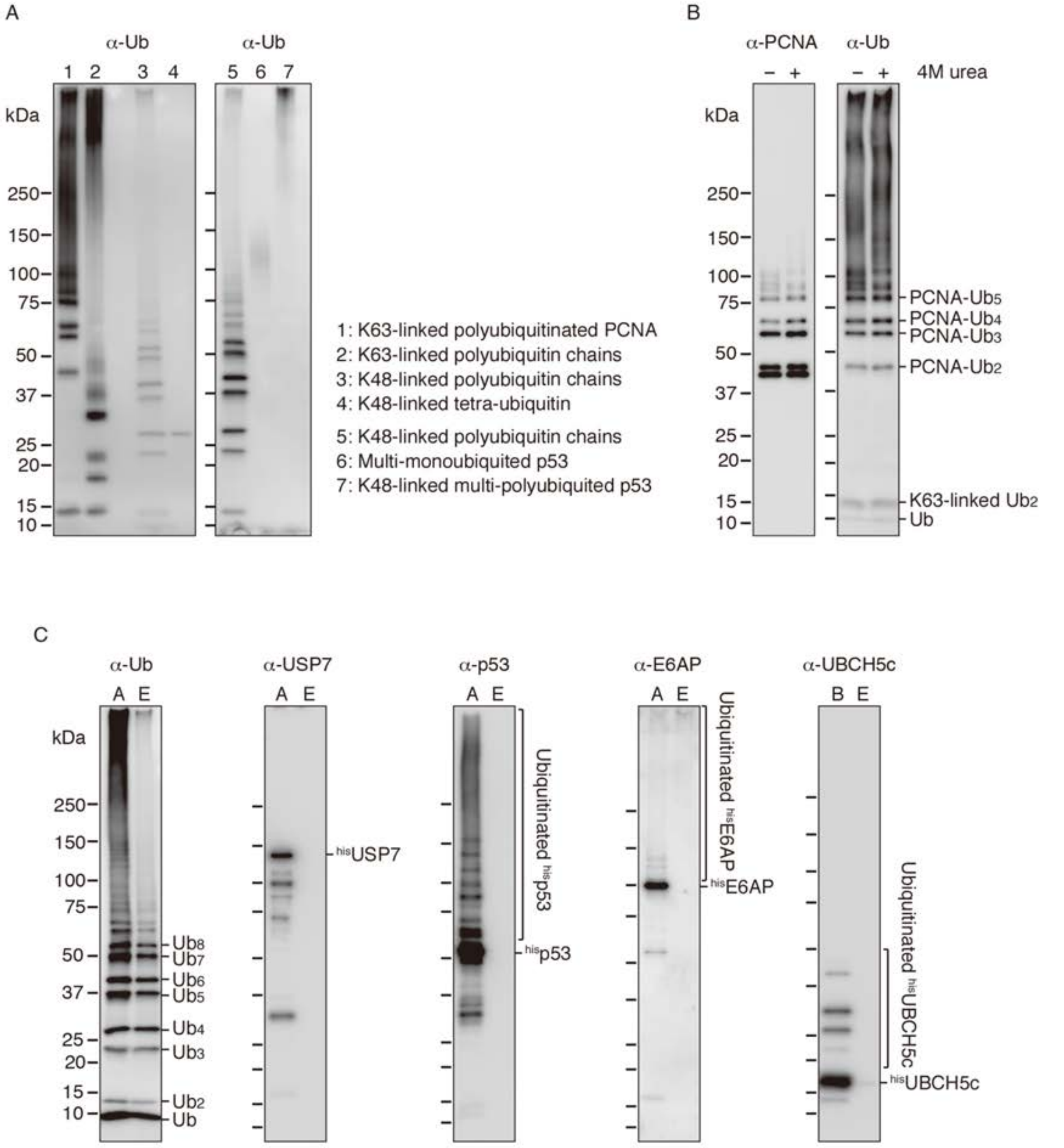

Supporting Figure S1

In vitro-generated substrates in this study. (**A**) Equivalent amounts used in the respective assays were subjected to western blotting with an anti-ubiquitin monoclonal antibody. Commercially available K48-linked tetra-ubiquitin was loaded as a control (lane 4). (**B**) Analysis of K63-linked polyubiquitinated PCNA as a substrate. The substrate was treated with SDS sample loading buffer in the presence or absence of 4 M urea before loading onto the gel, and then analyzed by western blotting with anti-PCNA or anti-ubiquitin monoclonal antibody. (**C**) Analysis of a mixture of K48-linked polyubiquitin chains as a substrate. The K48-linked polyubiquitin chains were released from polyubiquitinated ^{his}p53 by ^{his}USP7 digestion and subsequently purified using a Ni^{++}-chelating column. Western blot analysis with the indicated antibodies was used to examine the samples before loading onto the column before (B) or after (A) incubation with ^{his}USP7, as well as the fractions eluted from the column (E).

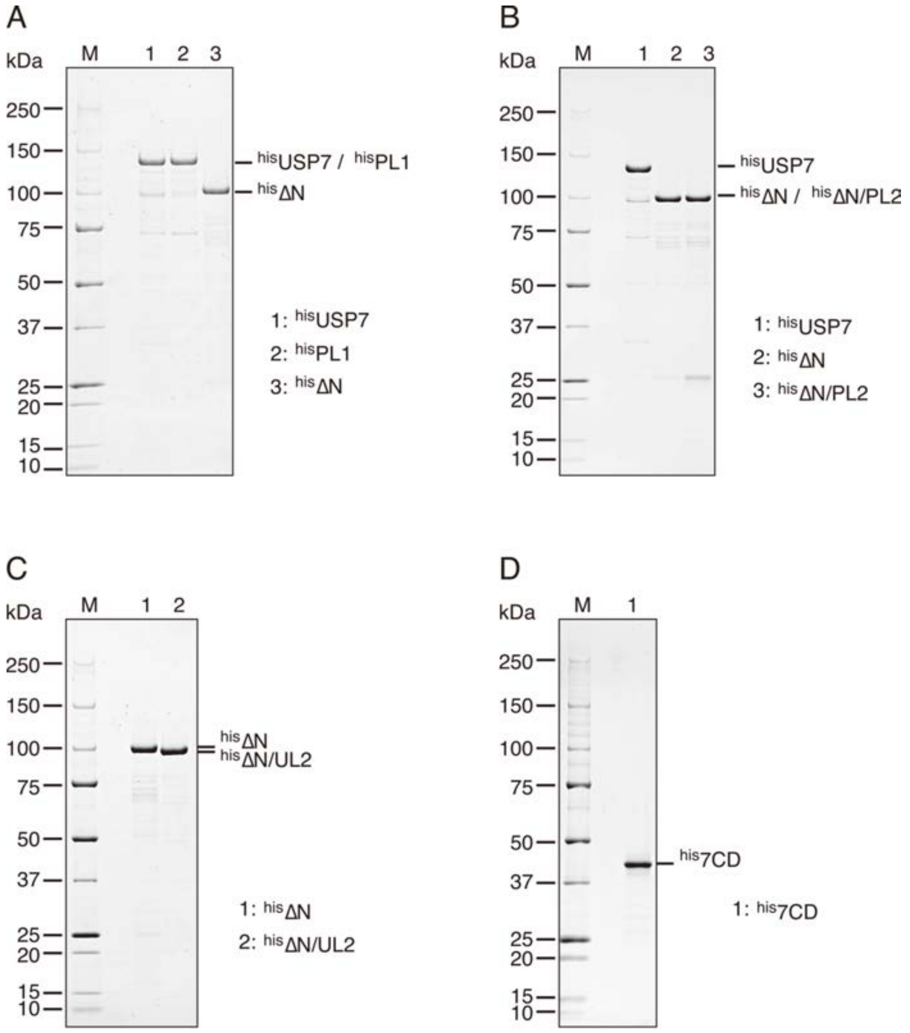

Supporting Figure S2

Purified proteins. The indicated proteins (500 ng each) were analyzed by SDS-PAGE and Coomassie Brilliant Blue (CBB) staining.

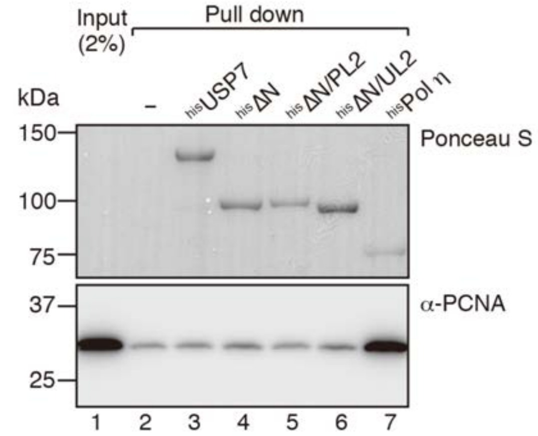

Supporting Figure S3

Pull-down assay of PCNA. The indicated proteins (lanes 3–7) or the buffer control (lane 2) immobilized on MagneticHis™ Ni particles was incubated with PCNA. After washing the beads, bound proteins were analyzed by western blotting. The membrane was stained with Ponceau S (upper panel). PCNA was detected with anti-PCNA antibody (bottom panel). Pol η is a positive control that binds to PCNA (lane 7).

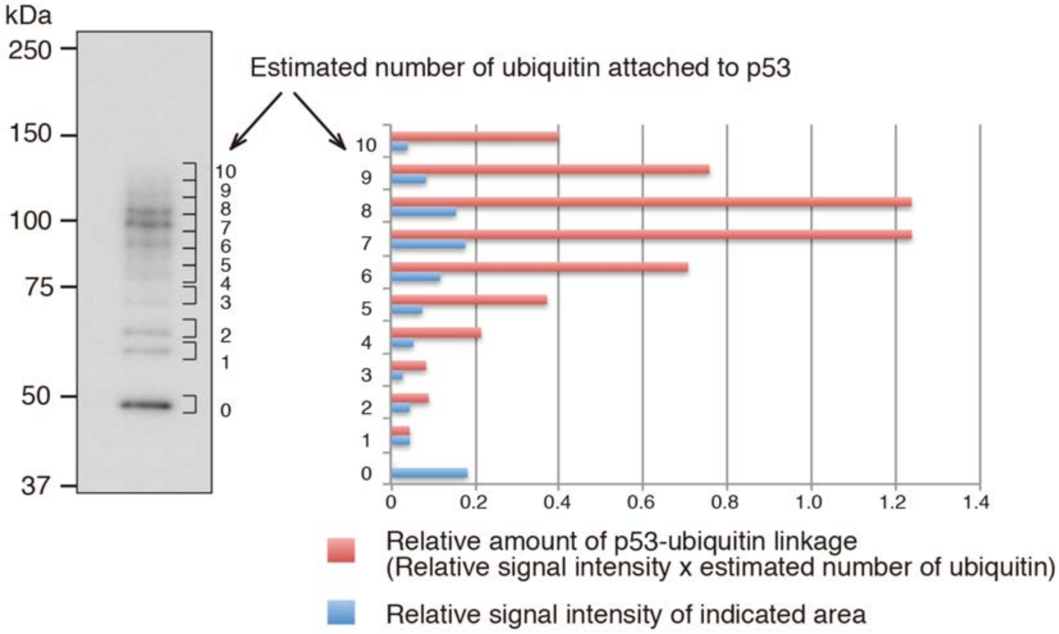

Supporting Figure S4

Estimation of the amounts of ubiquitin-p53 linkages in the multi-monoubiquitinated p53 substrate. The multi-monoubiquitinated p53 substrate was analyzed by western blotting with an anti-p53 antibody. The relative signal intensities of the indicated areas were measured (blue bars) and multiplied by the estimated number of ubiquitin molecules attached to p53 in each band (red bars). The sum of the values of red bars was 5.1-fold higher than the sum of the values of blue bars, indicating that an average of 5.1 molecules of ubiquitin are attached to one p53 molecule. The products of deubiquitination were analyzed in a similar manner to calculate the amounts of remaining ubiquitin molecules on p53.

Supporting Table S1. Comparison with published data on the catalytic activity of USP

USP	Substrate	Target linkage	Catalytic rate [24 nM] (min^{-1})	Reference
USP7	Poly-Ub-PCNA	Ub-PCNA	0.11 (±0.013)	This study
	Mono-Ub-PCNA	Ub-PCNA	0.068 (±0.0059)	This study
	Multi-Mono-p53	Ub-p53	0.042 (±0.0096)	This study
	K6-linked di-Ub	Ub-(K6)Ub	0.041 (±0.0058)	This study
			0.18[a]	(37)
	K11-linked di-Ub	Ub-(K11)Ub	0.050 (±0.0059)	This study
			0.093[a]	(37)
	K33-linked di-Ub	Ub-(K33)Ub	0.010 (±0.0017)	This study
			0.13[a]	(37)
	K48-linked di-Ub	Ub-(K48)Ub	0.016 (±0.0010)	This study
			0.17[a]	(37)
	K63-linked di-Ub	Ub-(K63)Ub	0.011 (±0.0018)	This study
			0.084[a]	(37)
	Ub-AMC	Ub-AMC	0.29[a]	(6)
			0.68–0.70[a, b]	(37,10)
			0.65[a]	(20)
PL1	Poly-Ub-PCNA	Ub-PCNA	0.092 (±0.0065)	This study
	Mono-Ub-PCNA	Ub-PCNA	0.047 (±0.0091)	This study
ΔN	Poly-Ub-PCNA	Ub-PCNA	0.25 (±0.027)	This study
	Mono-Ub-PCNA	Ub-PCNA	0.40 (±0.16)	This study
	Multi-Mono-p53	Ub-p53	0.054 (±0.016)	This study
	K48-linked di-Ub	Ub-(K48)Ub	0.019 (±0.0035)	This study
	K63-linked di-Ub	Ub-(K63)Ub	0.0093 (±0.0018)	This study
	Ub-AMC	Ub-AMC	0.051[a]	(6)
			0.53–0.72[a, b]	(37,10)
ΔN/PL2	Poly-Ub-PCNA	Ub-PCNA	0.086 (±0.0039)	This study
	Mono-Ub-PCNA	Ub-PCNA	0.15 (±0.030)	This study
	K63-linked di-Ub	Ub-(K63)Ub	0.0019 (±0.00014)	This study
ΔN/UL2	Poly-Ub-PCNA	Ub-PCNA	0.36 (±0.025)	This study
	Mono-Ub-PCNA	Ub-PCNA	0.94 (±0.10)	This study
	K63-linked di-Ub	Ub-(K63)Ub	0.020 (±0.0018)	This study
$USP2^{CD}$	Poly-Ub-PCNA	Ub-PCNA	0.092 (±0.026)	This study
	Mono-Ub-PCNA	Ub-PCNA	0.067 (±0.0067)	This study
	K48-linked di-Ub	Ub-(K48)Ub	0.023 (±0.0063)	This study
			0.10[a]	(38)
	K63-linked di-Ub	Ub-(K63)Ub	0.031 (±0.0030)	This study
	Ub-AMC	Ub-AMC	0.35[a]	(36)
			0.36[a]	(42)
			0.21[a]	(38)

[a] Velocity at 24 nM substrate was calculated from published kinetics parameters determined by the Michaelis-Menten equation, $v = (V_{max} \cdot [S])/(K_m + [S])$.

[b] Reported by the same group.
